# The human gut microbiome of athletes: metagenomic and metabolic insights

**DOI:** 10.1186/s40168-023-01470-9

**Published:** 2023-02-14

**Authors:** Federico Fontana, Giulia Longhi, Chiara Tarracchini, Leonardo Mancabelli, Gabriele Andrea Lugli, Giulia Alessandri, Francesca Turroni, Christian Milani, Marco Ventura

**Affiliations:** 1 Department of Chemistry, Life Sciences, and Environmental Sustainability, Laboratory of Probiogenomics, University of Parma, Parco Area Delle Scienze 11a, 43124 Parma, Italy; 2 GenProbio Srl, Parma, Italy; 3 Microbiome Research Hub, University of Parma, Parma, Italy

**Keywords:** Athletes, Microbiota, Metagenomics, Meta-Analysis, Enzyme, Synthase

## Abstract

**Background:**

The correlation between the physical performance of athletes and their gut microbiota has become of growing interest in the past years, since new evidences have emerged regarding the importance of the gut microbiota as a main driver of the health status of athletes. In addition, it has been postulated that the metabolic activity of the microbial population harbored by the large intestine of athletes might influence their physical performances. Here, we analyzed 418 publicly available shotgun metagenomics datasets obtained from fecal samples of healthy athletes and healthy sedentary adults.

**Results:**

This study evidenced how agonistic physical activity and related lifestyle can be associated with the modulation of the gut microbiota composition, inducing modifications of the taxonomic profiles with an enhancement of gut microbes able to produce short-fatty acid (SCFAs). In addition, our analyses revealed a correlation between specific bacterial species and high impact biological synthases (HIBSs) responsible for the generation of a range of microbially driven compounds such vitamin B12, amino acidic derivatives, and other molecules linked to cardiovascular and age-related health-risk reduction.

**Conclusions:**

Notably, our findings show how subsist an association between competitive athletes, and modulation of the gut microbiota, and how this modulation is reflected in the potential production of microbial metabolites that can lead to beneficial effects on human physical performance and health conditions.

Video Abstract

**Supplementary Information:**

The online version contains supplementary material available at 10.1186/s40168-023-01470-9.

## Introduction

In recent years, the increasing interest on the gut microbiota revealed how its relationship with the host is not limited to the intestinal environment but affects the entire human body across all the life stages, from birth to elderly [1, 2]. Stress and unbalanced diets are just two of the key drivers modulating the gut microbiota composition, shifting it towards a dysbiosis state, with potential negative impacts on systemic health [3–9]. On the contrary, a gut microbiota in homeostatic equilibrium is considered stable and able to maximize the beneficial interactions of the various members of the microbiota with the host, showing the capability of resisting external and internal influences [10].

While diet is one of the most impactful factors shaping the gut microbiota composition, physical activity can also modulate the gut microbiota through many mechanisms, such as the increased release of hormones and the redirection of blood from the gut to the skeletal muscles [11, 12]. In detail, the type of training, intensity, and duration of the physical activities impact the gut microbial population, ultimately altering its enzymatic potential responsible for systemic effects on the human host [12]. For example, studies concerning athletes have shown that they may be more susceptible to developing Inflammatory Bowel Diseases (IBD) [11, 13–16]. However, healthy athletes showed an increase in the production of short-fatty acid (SCFAs) for a greater energy intake, thereby contributing to host global metabolic efficiency [11, 17–19].

Remarkably, microbial SCFAs producers have been reported to generally possess a vast repertoire of metabolic pathways, not limited only to energy-related metabolism (i.e., short-chain fatty acid synthesis) but also including enzymes for amino acid and vitamin metabolism as well as for the synthesis of other by-products [20, 21].

However, despite the great scientific interest of this topic, the available scientific literature mainly focus on a limited range of well-known microbial taxa involved in the production of few metabolites, such as lactic acid and short-chain fatty acids. This is in contrast with the vast number of the microbial metabolic pathways encompassed by the gut microbiomes and therefore the high number of the potentially microbial produced health-active metabolites [3]. Thus, little is still known regarding the physiological mechanisms involving resident bacteria modulated by physical activity and their impacts on the host in terms of physical performances and systemic health. For this reason, it is becoming pivotal to gain insights into this intricate network of metabolic host-microbes’ interactions by analyzing in detail the gut microbiota composition in correlation with its genetic potential.

To delve into this intriguing area, in this study, we correlated physical activity metadata with taxonomical and microbial metabolic profiles of the gut microbiomes involving 185 athletes, 69 moderate athlete, and 166 controls (sedentary), using an in silico approach based on statistical analysis and correlations as well as hierarchical clustering and an optimized pipeline for metagenomic analysis.

## Results and discussion

### Metagenomic data selection and meta-analysis

In order to determine how physical activity can be associated to the modification in the composition of the gut microbiota and vice versa, the NCBI repository was screened for shotgun metagenomic samples related to the gut microbiota of professional athletes. Specifically, we used athletes’ metagenomics samples from multiple Bioprojects obtained from the same sequencing technology to avoid sampling-related bias. This screening resulted in the selection of a total of 185 metagenomic samples from a range of different sports fields, thus including sports with both high anaerobic and aerobic loads, such as marathon athletes as well as cyclists and rugby players [19, 22]. In addition, 164 metagenomic samples from healthy sedentary adults [23] were included in the study as a control group as well as 69 metagenomic samples of individuals identified as moderate athletes [24, 25].

Selected data led to a total of 418 shotgun metagenomic samples of athletes, sedentary and moderate athletes, supported with categorical (qualitative) physical activity-related metadata derived from their original studies (Table S1). To avoid data analysis biases, such data were re-analyzed following a common bioinformatic pipeline, i.e., METAnnotatorX2 [20]. All the metagenomic datasets showed an average of 3,100,774 reads per sample after Quality and Homo Sapiens filtering steps (Table S1).

### Taxonomic features associated with agonistic physical activity

The first step in the meta-analysis focused on performing descriptive analyses to correlate athletes, moderate athletes, and sedentary category (related to high, average and low physical activity) with the microbial taxonomic profiles, aiming to trace potential key microbial markers related to agonistic sport activity. Processing of all SRA samples through METAnnotatorX2 software (see the “Materials and methods” section for more details) allowed to retrieve of the taxonomic profiles of each analyzed metagenomic dataset with species-level accuracy [26] (Table S2). Furthermore, a hierarchical clustering analysis (HCL) was performed with an ideal number of centroids for the identification of the cluster that was extracted through a Silhouette analysis [27] (Figure S1).

**Fig. 1:**
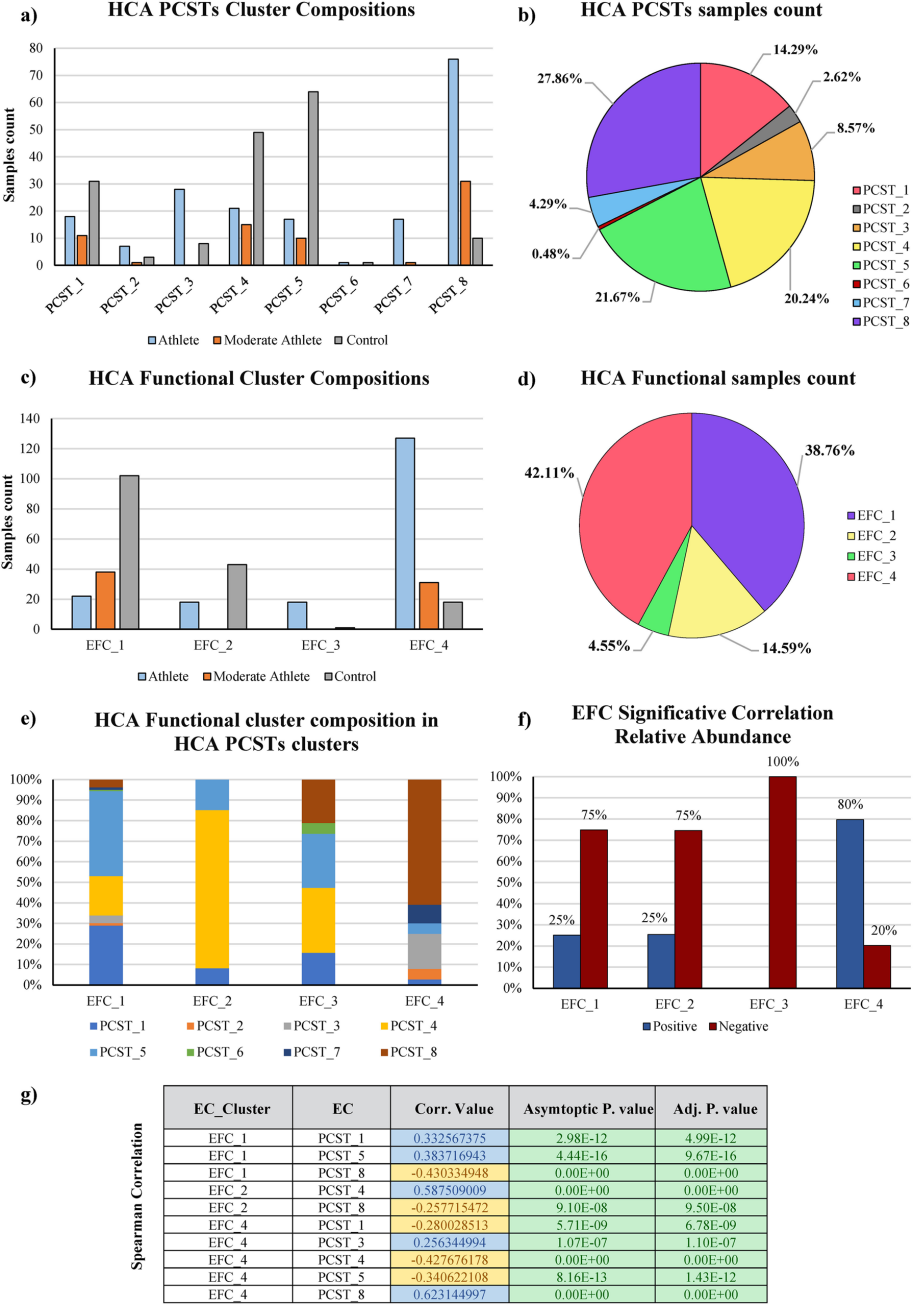
Samples subdivision between PCST and EFC clusters. In **a**, the PCST compositions in sample type (athlete, sedentary and moderate athlete) is reported, while in **b**, the total sample subdivision between the PCSTs is reported. Following the same logic, in **c**, the EFC compositions in samples type (athlete, sedentary, and moderate athlete) are reported, while in **d**, the total sample subdivision between the EFCs is reported. **e** The PCST distribution inside the EFC clusters. **f** The EFC correlation percentage with EC-Numbers. Finally, in **g**, the correlation score between EFCs and PCST clusters is reported

The HCL analyses identified a total of eight taxonomic clusters, named formally Physical activity level Community State Type (PCST) from PCST_1 to PCST_8, each characterized by a unique and recurring average bacterial composition profile (Table S3) (Figure S2). Notably, PCST_3, PCST_7, and PCST_8 represent clusters identified prevalently in the gut microbiomes of athletes and moderate athletes, and their sum represents 77.8%, 100%, and 91.5% of the predicted samples, respectively. In contrast, PCST_1, PCST_4, and PCST_5 were mainly found in the gut microbiomes of sedentary samples (144 out of 166) (Fig. 1a) (Table 1).

**Table 1:** PCSTs detailed samples subdivision

	**PCST_1**	**PCST_2**	**PCST_3**	**PCST_4**	**PCST_5**	**PCST_6**	**PCST_7**	**PCST_8**
**Athlete**	18	7	28	21	17	1	17	76
**Control**	31	3	8	49	64	1	0	10
**Moderate**	11	1	0	15	10	0	1	31
	**PCST_1**	**PCST_2**	**PCST_3**	**PCST_4**	**PCST_5**	**PCST_6**	**PCST_7**	**PCST_8**
**Athlete**	30.0%	63.6%	77.8%	24.7%	18.7%	50.0%	94.4%	65.0%
**Control**	51.7%	27.3%	22.2%	57.6%	70.3%	50.0%	0.0%	8.5%
**Moderate**	18.3%	9.1%	0.0%	17.6%	11.0%	0.0%	5.6%	26.5%

Notably, PCST_2 and PCST_6 contain less than 15 metagenomic samples, so they were excluded from our analysis because they are outliers, representing uncommon gut microbiota populations with limited statistical relevance (Fig. 1a, b) (Table 1).

**Fig. 2:**
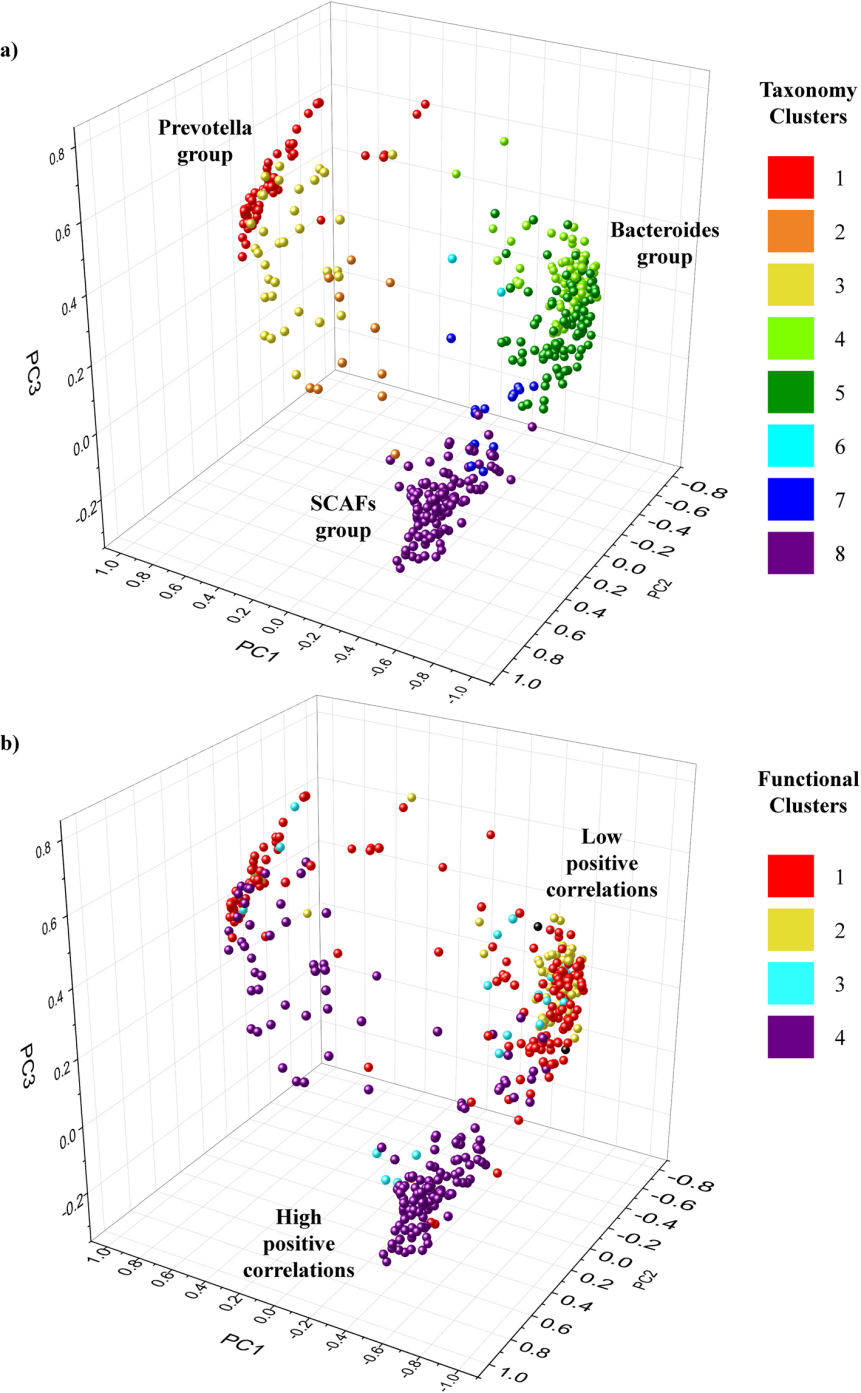
Beta diversity separations of samples based on their compositions and metadata. **a** The principal coordinate subdivision of the metagenomic samples, based on Bray–Curtis’s dissimilarity matrix of taxonomical composition, and subdivided for PCST clusters, with color scheme reported in legend. **b** The principal coordinate subdivision of samples, based on Bray–Curtis’s dissimilarity matrix of taxonomical composition, and subdivided for EFC clusters, with color reported in legend

Subsequently, we obtained the eigenvalues from the Bray–Curtis dissimilarity matrix, running a principal coordinate analysis (PCoA) to analyze the beta-diversity between the samples (Fig. 2).

Through the PCoA analysis, we found that the eight Physical activity Community State Type (PCST) sub-divide samples confirming the marked differences between the taxonomical composition of the different PCSTs (Fig. 2). Furthermore, these data revealed a substantial separation between athletes and sedentary individuals based on their gut microbiota taxonomical composition.

Remarkably, athlete-representative clusters identified based on the distribution of athlete’s samples (Fig. 1a) (Table 1), i.e., PCST_3, PCST_7 and PCST_8, shared a high occurrence of short fatty acid-producing microbial species (SCFAs producers), which distinguish them from the other taxonomic clusters analyzed (Mann–Whitney *U* adj. *P*-value < 0.05) (Table S3) (Table S4), thus confirming previous observations [19].

Bacterial SCFAs producers statistically associated to athletes’ samples (Mann–Whitney *U* adj. *P*-value < 0.05) (Table S4) include *Eubacterium rectale* (3.5 to 11.4% in average relative abundance), *Faecalibacterium prausnitzi* (4.5 to 8.2% in average relative abundance), and other unclassified *Faecalibacterium* species (4.5 to 9.5% in average relative abundance) (Figure S2) (Table S3). Additionally, it has been identified also other microbial species that are potentially involved in the synthesis of SCFAs, i.e., *Ruminococcus bromii* (0.4 to 3.4% in average relative abundance) but also putatively novel unclassified species of *Eubacterium* (1.3 to 2.4% in average relative abundance) and *Ruminococcus* species (1.5 to 3.4% in average relative abundance) (Mann–Whitney *U* adj. *P*-value < 0.05) (Table S4) (Figure S2) (Table S3). Altogether, the above-described bacterial taxa make up the “core” of SCFAs producers relating to athletes. Notably, PCST_3 also showed the presence of another SCFAs bacterial producer in addition to the above-mentioned “core,” i.e., *Prevotella*, and more specifically the dominant specie *Prevotella copri* (21.7%). Nevertheless, *Prevotella* is present also in the Sedentary-related PCST_1 (Kruskal–Wallis adj. *P*-value < 0.05) (Table S4) (Figure S2) (Table S3). *Prevotella* genus can act as an important microbial producer and consumer of SCFAs, but it has also been associated with various human inflammatory states [28, 29].

Intriguingly, all the PCST clusters containing the most prevalent SCFAs producers related to the genera *Faecalibacterium*, *Eubacterium*, and *Ruminococcus* were primarily identified in the gut microbiomes of athletes, thus reinforcing the previous notion that correlate SCFAs production to physical activity and the diet related to agonistic sports regimes.

Intriguingly, all PCST clusters containing the most prevalent SCFA producers of *Faecalibacterium*, *Eubacterium*, and *Ruminococcus* genera were identified primarily in the gut microbiomes of athletes, thus reinforcing the previous notion that SCFA production is higher in athletes compared to the other individuals (Fig. 1g) (Table S3).

### Functional analysis of potential-encoding enzymatic profiles

While SCFAs production has been extensively investigated for its impact on human health with a range of benefits [30–32], our current scientific understandings of the microbial metabolism leading to the production of secondary compounds involves thousands of enzymatic reactions encompassing catabolic and anabolic pathways, which may be responsible of the athletes’ performance and wellbeing. Hence, we performed a functional analysis of the 418 gut microbiomes aimed to identify the enzymatic pathways related to the production of chemical compounds that the scientific literature indicated as able to contribute to the human health by improving physical performances and quality of life. In this framework, METAnnotatorX2 was exploited to retrieve microbially based enzymatic profiles based on the MetaCyc database. Subsequently, a Bray–Curtis distance matrix was generated based on the enzymatic potential of each sample, in order to normalize the results and finally obtain a beta-diversity score (Table S2) (Table S5) that was employed for a hierarchical clustering (HCL) analysis.

We obtained a total of four enzymatic functional clusters (EFC) present in the pool of the analyzed samples, named EFC_1, EFC_2, EFC_3, and EFC_4 (Fig. 1) (Table S3). EFC_1 and EFC_4 represented the most populated clusters, comprising 38.8% and 42.1% of the total pool of samples. On the other hand, clusters EFC_2 and EFC_3 encompassed less frequent enzymatic profiles, including only 14.6 and 4.5% of the metagenomic samples, respectively (Fig. 1) (Figure S2). So, the latter clusters were excluded from further analysis, and we focused only on the most representative functional profiles.

Notably, EFC_4 was composed of 72% of athlete and 18% of moderate athlete, while EFC_1 included 63% of sedentary and 23.7% of moderate athlete (Table 2) (Fig. 1).

**Table 2:** EFCs detailed samples subdivision

	**EFC_1**	**EFC_2**	**EFC_3**	**EFC_4**
**Athlete**	22	18	18	127
**Moderate**	38	0	0	31
**Control**	102	43	1	18
	**EFC_1**	**EFC_2**	**EFC_3**	**EFC_4**
**Athlete**	13.58%	29.51%	94.74%	72.16%
**Moderate**	23.46%	0.00%	0.00%	17.61%
**Control**	62.96%	70.49%	5.26%	10.23%

Intriguingly, metagenomic samples belonging to moderate athletes were evenly distributed between EFC_1 and EFC_4, highlighting how non-intense or non-prolonged physical activity leads the samples to have in-between enzymatic profiles, an assumption validated by PERMANOVA analysis (adj. *P*-value < 0.001) (Table S6) (Fig. 1). Therefore, it can be extrapolated how EFC_4 is the most frequent enzymatic profile in the gut microbiome of athletes while EFC_1 is the most common in the gut microbiome of sedentary individuals. Thus, these findings support the strong association between athletes and the gut microbiota composition previously described (Fig. 1a) and highlight another association between athletes and the microbial-based enzymatic profiles (Fig. 1c) (Table 2).

Furthermore, we correlated the categorical data deriving from microbial enzymatic clusters (EFCs) with the taxonomic data (PCSTs) to obtain a complete overview of the taxonomic-enzymatic relationships. Such analyses highlighted that EFC_4 correlates with PCST_3, PCST_7, and PCST_8 (Spearman asymptotic adj. *P*-value < 0.005) (Fig. 1g), i.e., the clusters containing the SCFAs-producing bacteria “core” previously defined (Figure S2).

Intriguingly, 60.8% of EFC_4 is composed of metagenomic samples belonging to PCST_8, which is the taxonomical cluster with the highest presence of *Eubacterium rectale* as well as *Faecalibacterium prausnitzii* and other *Faecalibacterium* spp. (Figure S2) (Fig. 1).

Instead, the EFC_1 cluster correlates with PCST_1 and PCST_5 clusters (Spearman asymptotic adj. *P*-value < 0.005), mainly dominated by the genera *Prevotella*, *Bacteroidetes*, and *Alistipes*, with species such as *Prevotella copri*, *Bacteroides uniformis*, *Bacteroides stercoris*, and *Alistipes uniformis* (Figure S2) (Fig. 1g).

In addition, we further detailed each EFC-cluster’s association with each enzymatic reaction profiled, following the Enzyme Commission nomenclature (EC-Numbers) [33]. For this purpose, only those ECs displaying a prevalence > 10% were considered, for a total of 1604 EC numbers (Table S3) (Table S7). Unexpectedly, EFC_4 displays 725 of positive correlations (80% of its total statistically significative correlation, Spearman asymptotic adj. *P*-value < 0.05) with the retained ECs, showing a large gap compared to the EFC_1, which on average showed a total of only 79 positive correlations (25% of its total statistically significative correlation) (Table S7) (Fig. 1f). These findings, clearly corroborate what preliminary observed in a previous study [34] encompassing a small cohort of individuals analyzed with a less accurate metagenomic approach such as the 16S rRNA gene microbial profiling. Remarkably, the shotgun metagenomic approach allowed us also to explore in detail the metabolic relevance of enzymatic reactions positively correlated with physical activity.

### Characterization of microbial biosynthetic metabolisms associated with physical activity

The two main enzymatic clusters, i.e., EFC_1 and EFC_4, were also exploited to investigate those enzymes involved in the anabolism of key metabolites known to impact on host’s health by the recent scientific literature [35, 36]. In this context, a selection of EC numbers was manually investigated for their possible relevance and were named high biological impact synthases (HBIS) (Table S8). Notably, these ECs were selected based on information reported in the MetaCyc database and cited literature data [35, 36] (Table S8).

A comparison of the enzymatic profiles of HBIS between the two groups revealed 66 HBIS positively correlated with cluster EFC_4 (representing the most common enzymatic profile of athletes) and only 10 with cluster EFC_1 (representing the most common functional profile of sedentary individuals) (Table S8). Therefore, the EFC_1 enzyme cluster displays a lower HBIS production potential than EFC_4, highlighting how the microbiota of athletes can potentially encode for a much wider range of microbial metabolites with an important impact on health and physical performance.

In detail, between the 14 HBIS positively related to EFC_1 there are EC related mainly to vitamin biosynthesis, but also related to flavodoxin precursor, a well-known phosphoantigen also required by many pathogens to survive [37, 38] (Table 3).

**Table 3:** HBIS positively correlated to EFC_1 and manually identified with MetaCyc database

Spearman correlation (adj. *P*-value < 0.05)					
**EC name**	**EC number**	**EFC_4**	**EFC_1**	**Related product effects**	**Ref**
Isochorismate synthase	5.4.4.2	− 0.541970	0.311153	Production of the precursor of vitamin K_2_	[39]
1,4-dihydroxy-2-naphthoyl-CoA synthase	4.1.3.36	− 0.543416	0.316668	Production of the precursor of vitamin K_2_	[40]
Quinolinate synthase	2.5.1.72	-	0.266977	Precursor of niacin and indirectly of vitamin B_3_	[41]
(E)-4-hydroxy-3-methylbut-2-enyl-diphosphate synthase	1.17.7.3	− 0.598568	0.333534	Production of phosphoantigen	[37]

In contrast, among the 73 positive correlations between EFC_4 and HBIS, we extracted and focused on eight enzymes related to the enhancement of sports performance and the increase of life span through the reduction of the onset of cardiovascular diseases and tumors. Among the enzymes selected, there is also an enzyme involved in the production of the heme group and therefore in the regeneration and production of new blood cells (Table S8) (Table 4).

**Table 4:** HBIS positively correlated to EFC_4 and manually identified with MetaCyc database

Spearman correlation (adj. *P*-value < 0.05)					
**EC name**	**EC number**	**EFC_4**	**EFC_1**	**Related products effects**	**Ref**
Spermidine synthase	2.5.1.16	0.764993	− 0.340411	Reduction in mortality due to cardiovascular disease	[42, 43]
Porphobilinogen synthase	4.2.1.24	0.590898	-	Heme biosynthesis	[44]
Mycothiol synthase	2.3.1.189	0.771842	− 0.319983	Antibacterial and antitumoral properties	[45, 46]
Hydrogenobyrinic acid a,c-diamide synthase	6.3.5.9	0.642982	− 0.284009	Coenzyme B12 (cobalamin) biosynthesis	[47, 48]
Cystathionine gamma-synthase	2.5.1.48	0.712590	− 0.256458	Energy metabolism, muscle performance, antioxidant	[49–54]
Glutamate synthase (NADPH)	1.4.1.13	0.623222	-	Excitatory neurotransmitter, homocysteine balance	[55–58]
Asparagine synthase	6.3.5.4	0.645870	-	Excitatory neurotransmitter, homocysteine balance	[55–58]
Glutaminyl-tRNA synthase	6.3.5.7	0.768508	− 0.284009	Excitatory neurotransmitter, homocysteine balance	[55–58]

Additionally, EC numbers related to the production of sulfur amino acids and molecules like glutathione (GSH) and taurine were correlated positively with EFC_4, potentially enhancing the reduction of oxidative-cellular damage and boosting muscular performance (Table S8) (Table 4).

**Fig. 3:**
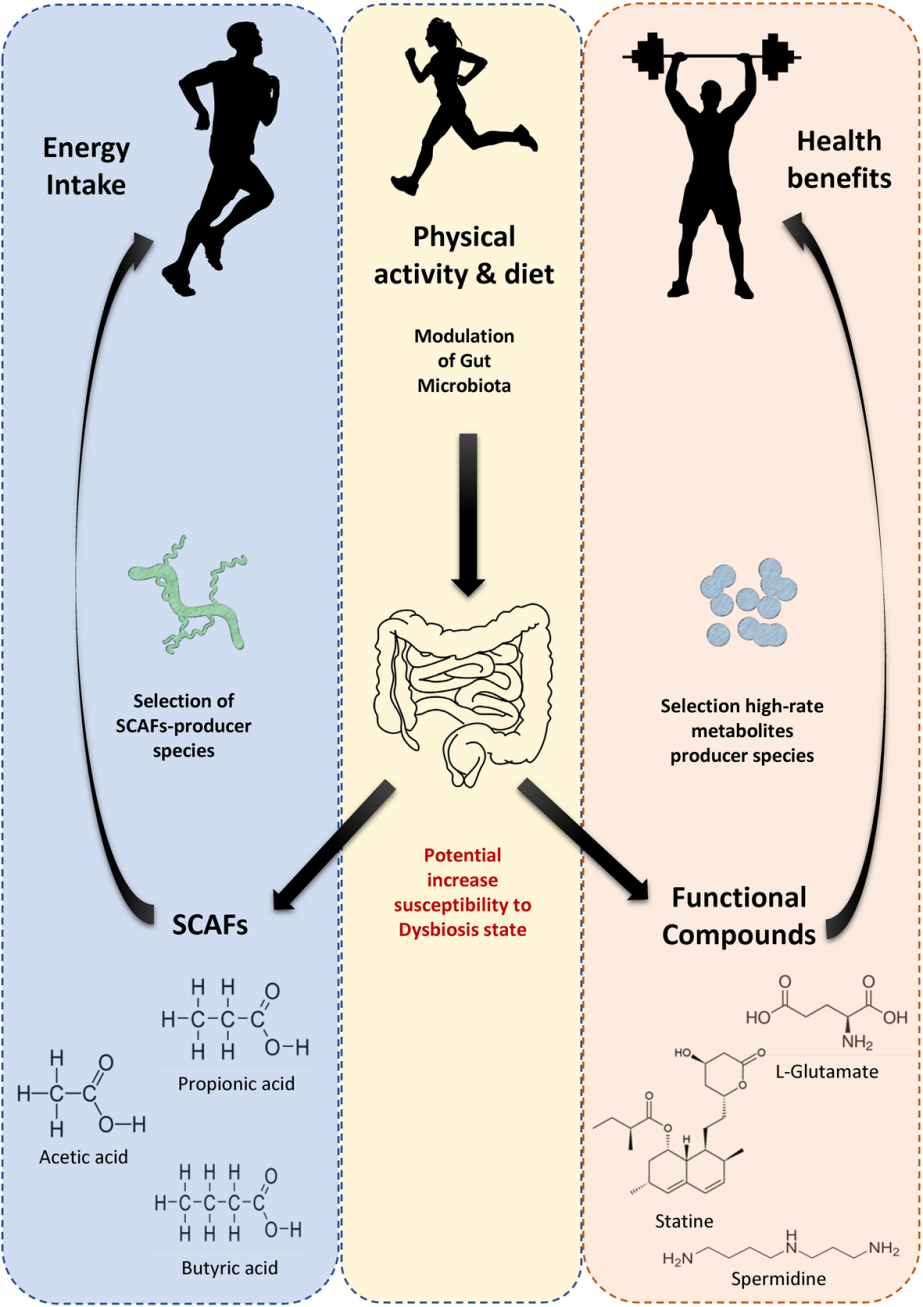
Schematic representation of the project aims and key points. The modulation effect that physical activity can exert on gut microbiota and vice versa the effect that gut microbiota can exert on human health and performance. Some of the main compounds produced by SCFAs producers are reported with name and structural formula

Altogether, these results evidenced that the microbiomes of the samples belonging to athlete’s category are characterized by a higher abundance of biosynthetic enzymes involved in the production of a wide range of compounds (Fig. 3).

### Associations between HBIS and core microbial taxa

We performed a taxonomic EC back-tracking analysis to investigate further the main bacterial taxa responsible for the above-reported enzymatic reactions associated with physical activity. This approach aims to identify the bacterial species that can potentially produce the nine HBIS positively correlated to the EFC_4 above-discussed.

As expected, the “core” of SCFAs producers found in athlete metagenomic samples, such as *Faecalibacterium prausnitzii*, *Eubacterium rectale*, and *Blautia wexlerae* and a set of minor representative species of *Faecalibacterium*, *Eubacterium*, *Ruminococcus*, and *Blautia* genera act as major microbial producers of the nine enzymatic reactions previously highlighted as possessing a high putative health interest in the EFC_4 cluster (Table S9). In detail, six EC classes (EC 6.3.5.9, 6.3.5.7, 4.2.1.24, 2.5.1.16, 6.3.5.4, and 2.3.1.189) resulted to be produced primarily by the above-identified “core” of SCFAs producers. Moreover, EC 1.4.1.13, a glutamate synthase (NADPH), was found to be produced more specifically by *Faecalibacterium prausnitzii*, *Eubacterium rectale*, and other *Faecalibacterium* species (Table S9). In contrast, EC 2.5.1.48, which encompasses a cystathionine gamma-synthase, was predicted to be produced by a more variegated number of species, including *Anaerostipes*, *Ruminooccus*, and *Coprococcus* species, along with *Bifidobacterium adolescentis* (Table S9).

Intriguingly, these data revealed clear associations between specific functional features and microbial taxa harbored by the intestinal environment of athletes.

## Conclusion

With the purpose of analyzing the intricate relationships between the gut microbiome and athletes’ related lifestyle (multifactorial metadata including training, diet, and stress), we statistically analyzed 418 metagenomic samples divided into athlete, sedentary, and moderate athletes. As a result of taxonomical profiling, we identified a correlation between gut microbial profiles and athlete’s category, as evidenced by a recurrent microbial pattern defined primarily by SCFAs microbial producers including *Faecalibacterium*, *Eubacterium*, *Blautia*, and *Ruminococcus* species, which are statistically associated to athletes’ samples (Table S4).

In addition, subsequent functional analysis showed the presence of two major enzymatic functional clusters (EFCs), one strongly associated with the presence of sedentary individuals and one with athletes, thus corroborating the differences previously seen at species-taxonomical level between the two types of samples (athletic and sedentary subjects). Intriguing, the EFC related to athletes was positively linked to 752 enzymes (EC numbers) and 73 high biological impact synthases (HIBS), a subset of manually identified biosynthetic reactions. In contrast, the EFC related to sedentary resulted in being positively linked only to 105 EC numbers and 14 HBIS, highlighting the reduced ability of sedentary’ gut microbiota to affect the host health through the production of secondary metabolites. Furthermore, the correlation of the enzymatic potential with species-level microbial profiles evidenced how additional microbial taxa may be implicated in the biosynthesis of compounds of high biological interest.

Remarkably, these data highlighted how the athletes’ related lifestyle represent a multifactorial ecological pressure that modulate the gut microbiota, reshaping it in favor of bacterial species with a higher enzymatic potential impacting the host’s health and muscular performances. Additionally, all these results pointed out how the bacterial species commonly considered core SCFAs producers are also implicated in the production of a much wider and variegated range of potentially high functional impact molecules, which will require a precise characterization in future population studies.

## Materials and methods

### Metagenomic sample collection

A set of 418 shotgun metagenomic data were retrieved from the National Center of Biotechnology Information (NCBI) Sequence Read Archive (SRA) database. The terms used to inspect the scientific literature include athlete, gut microbiota, IBD, SCFA, sedentary, performance, and physical activity. For the selection of the optimal Bioprojects for this study, we used various criteria, such as the selection of healthy samples, the sequencing technology, the minimum number of reads available, and finally the completeness of the metadata regarding athlete and sedentary categories. All Bioprojects have been manually checked to ensure that minimum criteria were met. In detail, each metagenomic dataset possess a minimum of 10,000 reads, according to the minimum sequencing depth required to METAnnotatorX2 for obtaining high-quality taxonomical profiles [26]. Accordingly, we collected shotgun metagenomics sequences and associated metadata from six different studies (PRJEB15388, PRJEB28338, PRJEB32794, PRJNA472785, PRJNA305507, PRJEB20054). The selection of six different sources (Bioprojects) of raw data sequenced through Illumina technology allowed reduced selection bias. Additionally, this selection was performed to obtain a comparable number of samples between athletes and controls. In detail, 185 samples corresponded to athlete gut microbiomes, 69 to moderate athlete, and 164 were from healthy sedentary individuals (Table S1). The athletes and the sedentary categories were defined by metadata originating from their original scientific articles and Bioprojects. Moderate athletes instead refer to athletes who have performed competitive activity only for a short time window (high school athletes) or without reaching the higher categories [therefore CAT 1 (semi-professional) vs. PRO athletes]. Specifically, between the 69 moderate athletes’ samples were included time-longitudinal samples belonging to Bioprojects PRJNA472785 and PRJNA305507 to increase the robustness of the analysis regarding the group composed by moderate athletes. Thus, the small group of moderate athletes was used to compare and validate the distribution of the two main analysis groups (athletes and controls). Additional metadata regarding physical status, type of sport performed, and other miscellaneous are reported along with the SRA name in Table S1. All available metadata regarding the metagenomic samples (mainly athletic and sedentary designation) were retrieved from the Bioprojects related to the samples.

### Metagenomics data processing, taxonomic profiling, and functional analysis

Each metagenomic datasets were filtered to remove reads with a base sequence quality of < 25 (score obtained from FastQC software for Illumina sequencing) and to retain reads with a length of > 149 bp. Taxonomic and functional profiling of reads resulting from quality and *Homo sapiens* filtering was performed with the METAnnotatorX2 bioinformatics platform [26, 59]. Within the METAnnotatorX2 pipeline, MegaBLAST [60] was employed for taxonomic classification of each metagenomic read, using a curated non-redundant sequence database of genomes retrieved from NCBI servers and manually selected. The generation of the taxonomical database was reported in detail by Milani et al. [26] and periodically updated (every 6 months). Reads with a nucleotide identity of > 94% to reference genomes are classified at the species level, while reads with a lower percentage identity are classified at the genus level as undefined species. The functional enzymatic classification of each metagenomic read was performed through DIAMOND [61], employing a curated non-redundant sequence database of EC number sequence created employing the MetaCyc database [62]. DIAMOND parameters used for this analysis were as default chosen by the METAnnotatorX2 pipeline using up to 5,000,000 reads (–query-cover 80, –evalue 0.00000001, and –max-target-seqs 1). Taxonomic EC back-tracking analysis was performed using METAnnotatorX2 -x ec_taxonomy function. This function allowed to retrieve the bacterial species related to the production of a selected list of enzymatic codes.

For the analyses that required the use of R software, version R-4.1.2 was used, along the version RStudio-2021.09.2–382 of R Studios and rtools40v2-x86_64 of rtools.

Similarities between samples (beta-diversity) were calculated using the Bray–Curtis distance matrix based on species relative abundance, using the vegdist function (from vegan_2.5–7) on R-Studios (RStudio Team (2020). RStudio: Integrated Development for R. RStudio, PBC, Boston, MA URL.). The range of similarities is calculated between values 0 and 1. PCoA representation of beta-diversity was performed using ORIGIN 2021b (https://www.originlab.com/2021).

In the PCoA, each dot represented a sample, distributed in tridimensional space according to its bacterial composition, i.e., eigenvalues scores. The hierarchical clustering analysis (HCA) of samples, performed on ORIGIN 2021b, was achieved employing Bray–Curtis matrix using Pearson correlation as a distance metric and the sum square of distances and furthest neighbor for clustering methods. The optimal number of clusters was defined through a Silhouette analysis [27] performed on ORIGIN 2021b. The data obtained was represented by a vertical dendrogram.

### Statistical analysis

ORIGIN 2021b (https://www.originlab.com/2021), IBM SPSS statistics software (version 25) (www.ibm.com/software/it/analytics/spss/) and R-Studios were used to compute statistical analyses. PERMANOVA analyses were performed on R-studios using 999 permutations to assess *p*-values for population differences in PCoA analyses. In detail, input data was preprocessed and transformed in a Bray Curtis dissimilarity matrix with vegdist function (from vegan_2.5–7), and the PERMANOVA analysis was performed with adonis2 package (from vegan_2.5–7). Non-parametric Kruskal–Wallis’s test was performed on SPSS software using PCSTs subdivision as group criteria. In addition, a pairwise post hoc analysis was performed for the Kruskal–Wallis’s analysis, using Bonferroni correction for the FDR adj. *p* value. Non-parametric Mann–Whitney *U* test was performed on SPSS software using PCST_1, PCST_4, and PCST_5 as group 1 and PCA_3, PCST_7, and PCST_8 as group 2. Spearman correlation was performed with rcorr function (from Hmisc_4.6–0), and only statistical significative results with correlation score greater than 0.25 or minor of − 0.25 were retained. The eigenvalues were retrieved from the Bray Curtis dissimilarity matrix with the use of prcomp function (from base package stats) and the get_pca function (from factoextra_1.0.7). All the raw *p*-value with the exclusion of Kruskal–Wallis’s pairwise post hoc were subjected to FDR correction using Benjamini-Hochberg [63] approach on R-studios through p.adjust function (from base package stats).

## Supplementary Information

**Additional file 1: Table S1.** Samples metadata summary.

**Additional file 2: Table S2.** Relative abundance profiles of bacterial species for each sample (Sheet_1). Relative abundance profiles of EC Numbers for each sample (Sheet_2).

**Additional file 3: Figure S1.** a) Silhouette analysis and HCL circular tree based on taxonomical data and subdivided in a number of clusters equal to the identified centroids from the silhouette analysis through an HCA analysis. b) Silhouette analysis and HCL circular tree based on enzymatic data and subdivided in a number of clusters equal to the identified centroids from the silhouette analysis through an HCA analysis.

**Additional file 4: Figure S2.** PCSTs bacterial species composition represented through a Bar-Plot representation.

**Additional file 5: Table S3.** Average relative abundance and prevalence analysis of bacterial species inside each PCSTs (Sheet_1). Average relative abundance and prevalence analysis of EC Numbers inside each EFCs (Sheet_2).

**Additional file 6: Table S4.** Mann-Whitney U statistical analysis between PCSTs related to athletes and sedentary samples (Sheet_1). Statistical Kruskall-Wallis analysis of bacterial species between PCST clusters (Sheet_2).

**Additional file 7: Table S5.** Bray-Curtis dissimilarity matrix based on taxonomical data (Sheet_1). Bray-Curtis dissimilarity matrix based on functional data (Sheet_2).

**Additional file 8: Table S6.** PERMANOVA results based on Bray-Curtis Functional profiles.

**Additional file 9: Table S7.** Full Spearman correlation analysis result of all the EC numbers Vs. all the EFC (1-4) identified.

**Additional file 10: Table S8.** Correlation analysis results of all the HBIS against EFC_1 and EFC_4.

**Additional file 11: Table S9.** EC Back-tracing report of the extracted nine HBIS manually analyzed on MetaCyc and related to EFC_4.

## Data Availability

All data can be retrieved from NCBI SRA repository through their SRA Accession Number reported in Table S1.
