# Supplementary figures and images for: The human gut microbiome of athletes: metagenomic and metabolic insights
Source: Microbiome. 2023 Feb 14;11:27. doi: 10.1186/s40168-023-01470-9 (PMC9926762; doi:10.1186/s40168-023-01470-9)

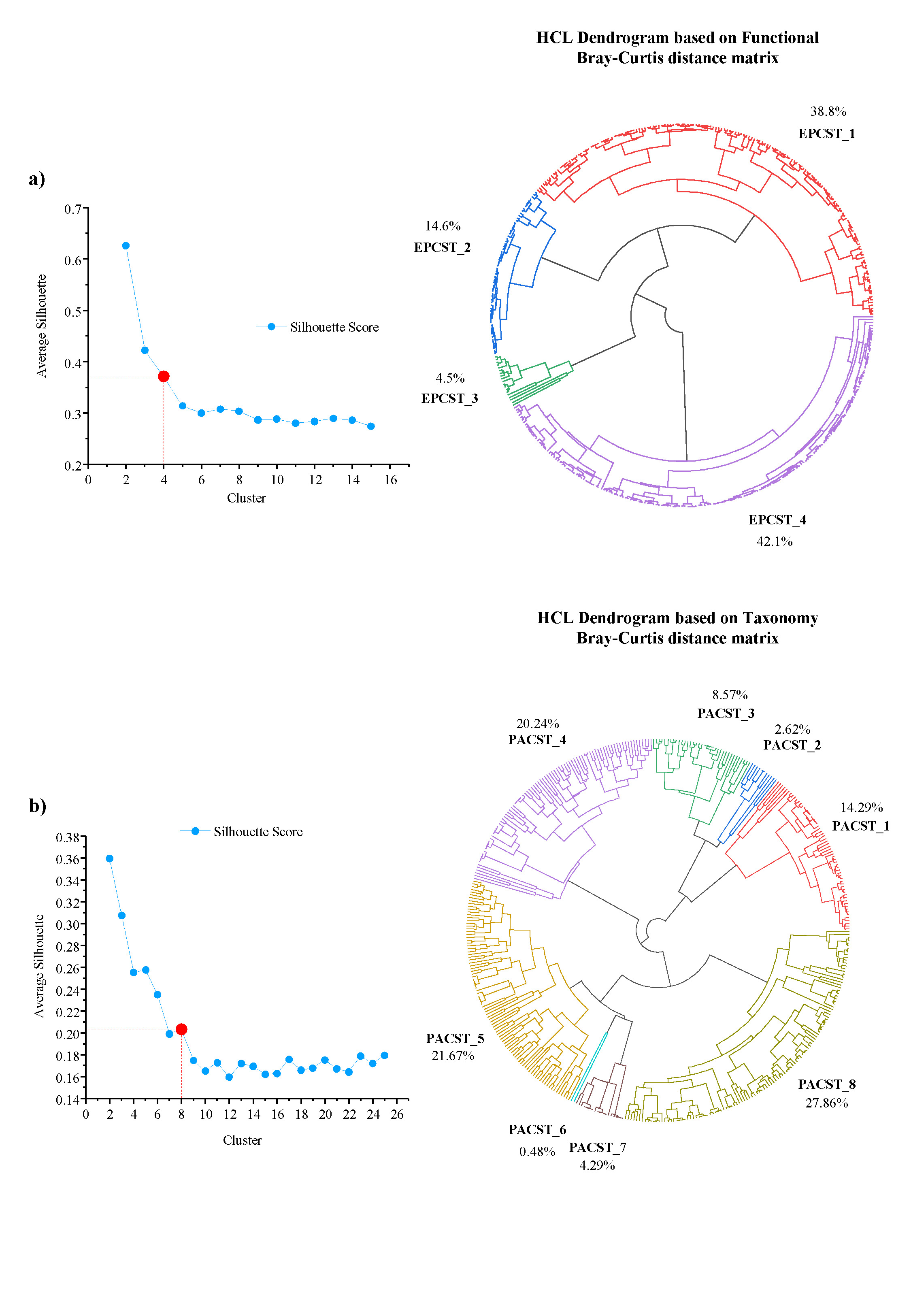

Supplement: Supplementary file 4 — Additional file 3: Figure S1. a) Silhouette analysis and HCL circular tree based on taxonomical data and subdivided in a number of clusters equal to the identified centroids from the silhouette analysis through an HCA analysis. b) Silhouette analysis and HCL circular tree based on enzymatic data and subdivided in a number of clusters equal to the identified centroids from the silhouette analysis through an HCA analysis. [file 40168_2023_1470_MOESM3_ESM.jpeg]

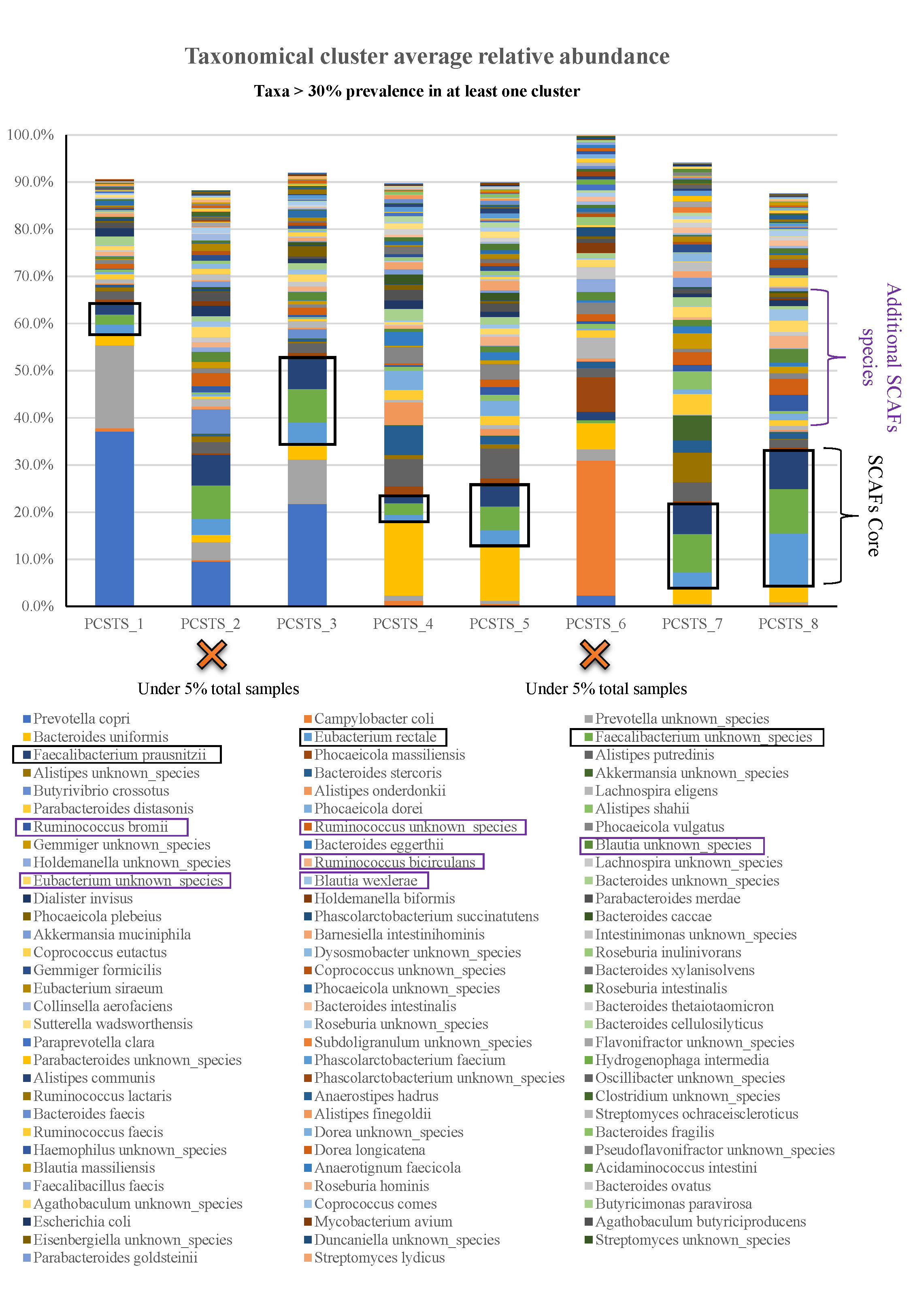

Supplement: Supplementary file 5 — Additional file 4: Figure S2. PCSTs bacterial species composition represented through a Bar-Plot representation. [file 40168_2023_1470_MOESM4_ESM.jpeg]
